# Fostering inclusive participation in non-invasive neurotechnology research: a community-based study with an underrepresented population

**DOI:** 10.3389/fnhum.2026.1846343

**Published:** 2026-07-29

**Authors:** Mohamed A. A. Mohamed, Caitlin Illingworth, Mian Kou, Layla Kouara, Sahra Abdi, Muse Jama, Hesam Olya, Lise Sproson, Dan Blackburn, Mahnaz Arvaneh

**Affiliations:** 1 School of Electrical and Electronics Engineering and Neuroscience Institute, University of Sheffield, Sheffield, United Kingdom; 2 School of Medicine and Population Health, Sheffield Institute for Translational Neuroscience (SITraN), University of Sheffield, Sheffield, United Kingdom; 3 Israac Somali Community Association, Sheffield, United Kingdom; 4 Management School, University of Sheffield, Sheffield, United Kingdom; 5 National Institute for Health and Care Research (NIHR) HealthTech Research Centre Long Term Conditions, Devices for Dignity, Sheffield, United Kingdom

**Keywords:** community engagement, community-based research, ethnic minority participation, inclusive research, neurotechnology

## Abstract

Despite the growing role of neurotechnologies in neuroscience and clinical care, Black African communities remain critically underrepresented in the UK research and clinical trials. Historical lack of diversity in neurotechnology studies raises important concerns regarding accessibility, trust, cultural appropriateness, and whether research practices and emerging technologies adequately reflect the needs and lived experiences of underrepresented communities. This study presents, to our knowledge, the first comprehensive community-led, mixed-methods investigation into barriers and enablers of participation in non-invasive neurotechnology research within a UK Somali community. Moving beyond laboratory-based engagement approaches, we co-designed and delivered a three-workshop programme covering neuroimaging (EEG/fNIRS), brain stimulation, and reflective co-creation, hosted within a trusted community center with gender-matched facilitation and interpreter support. The study focused on identifying practical, cultural, and structural barriers affecting participation in neurotechnology research and on co-producing inclusive adaptations to improve accessibility and acceptability. Thirty community members (balanced by gender) took part in workshops, focus groups, and interviews; a subset (*n* = 10) completed optional EEG/fNIRS sessions on site. A structured thematic analysis (Gioia approach) integrated workshop notes, questionnaires, and interviews, with member reflections in Workshop 3 providing respondent validation. The engagement design explicitly accommodated cultural-religious needs (privacy, prayer times, gender matching) and supported language inclusion. Four barrier domains were identified: (1) structural/socioeconomic constraints, (2) cultural and religious requirements, (3) limited awareness and misconceptions, and (4) trust/accountability gaps. Co-produced adaptations (venue choice, staffing, scheduling, multilingual materials, and prototype hardware) directly addressed these barriers and enhanced acceptability. The study delivers an evidence-informed framework for inclusive, community-centered neurotechnology research, advancing equitable participation while maintaining methodological and technical integrity.

## 1 Introduction

Neurotechnology refers to technical systems and methods that enable direct interaction between external devices and the human nervous system. By recording or modulating neural activity, these technologies offer new opportunities for diagnosing and treating neurological and psychiatric conditions, while also expanding therapeutic possibilities (Berger et al., 2008; Biasiucci et al., 2019; Williams et al., 2013; Chaudhary, 2025; Wagner et al., 2018; Noachtar and Rémi, 2009; Müller and Rotter, 2017; Nitsche et al., 2009; Kim et al., 2021).

Despite rapid advances in the field, many minoritized and underrepresented communities remain excluded from neuroscience and neurotechnology research (Armstrong, 2024; Green et al., 2022). This lack of representation raises important concerns about inclusivity, accessibility, trust, and whether emerging technologies adequately reflect the needs and lived experiences of diverse populations. Researchers continue to under-recruit participants from minoritized groups, contributing to disparities in both research findings and clinical applications (Armstrong, 2024). These disparities are further compounded by practical challenges in signal acquisition and device setup across diverse populations, as well as broader barriers to participation and accessibility (Brown et al., 2023; Yücel et al., 2024). Together, these issues highlight the need for more inclusive and community-informed research practices.

These concerns are particularly evident for Black populations, who remain severely underrepresented in neuroscience research. In the UK, for example, large-scale neuroimaging studies such as the UK Biobank include over 95% White participants, reflecting a wider global pattern of exclusion. A review of neuroscience clinical trials conducted between February 2016 and February 2021 across 47 countries found that only 1.6% of more than 8,000 participants identified as Black (Rutten-Jacobs et al., 2024). This systemic underrepresentation undermines both the scientific validity and the inclusivity of emerging neurotechnologies. When these technologies are not tested or adapted for diverse users, they may lead to discomfort, poor data quality, and reduced user acceptance, particularly for individuals with phenotypic traits not considered in standard device design (Brown et al., 2023; Yücel et al., 2024).

The drivers of this underrepresentation are multifaceted, involving both structural and socio-cultural barriers. Historical mistrust in research, shaped by past unethical practices, continues to influence perceptions and willingness to participate, particularly among Black and other minoritized populations (George et al., 2014). Language and cultural mismatches, lack of accessible information, and limited community collaboration further discourage participation (Hall et al., 2022; George et al., 2014; Algahtani et al., 2018). Studies have also shown that many individuals are reluctant to participate due to unclear research intentions, concerns about how their data will be used, and a lack of feedback or perceived benefit from involvement (Matshabane, 2021; Peebles et al., 2023). Additionally, stigma around brain research—often linked to fears of being labeled as mentally ill or cognitively impaired—can deter individuals from engaging in studies involving neurotechnologies (George et al., 2014; Murphy and Thompson, 2009).

**Figure 1:**
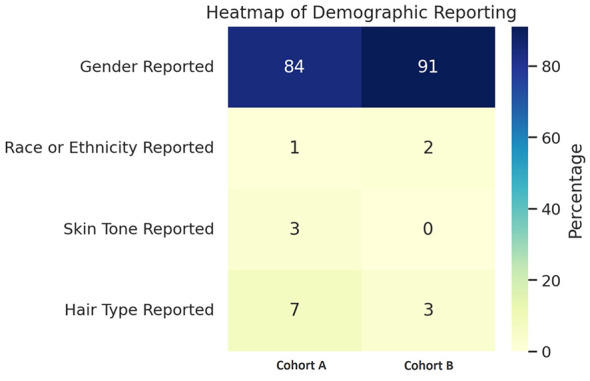
Demographic attribute reporting in two cohorts of neuroimaging studies (Kwasa et al., 2023). Cohort A includes 90 empirical studies from leading biomedical optics journals, showing frequent reporting of gender but limited reporting of race/ethnicity, skin tone, and hair type. Cohort B, comprising 87 studies from prominent neuroscience journals, reflects similar patterns—high reporting of gender with minimal attention to other key demographic variables.

Compounding these issues is the ongoing failure of neuroscience and neurotechnology literature to report key demographic attributes. The reporting of demographic attributes in two cohorts of neuroimaging studies further highlights these disparities (Figure 1). An analysis of 90 empirical studies published in leading biomedical optics journals revealed that gender is frequently reported, while race/ethnicity, skin tone, and hair type are largely absent (Kwasa et al., 2023). A similar pattern was found across 87 articles from prominent neuroscience journals, with high rates of gender reporting but minimal documentation of other critical characteristics (Kwasa et al., 2023; Sterling et al., 2022). When demographic factors such as race, skin tone, and hair type are not reported or considered, neurotechnologies risk being optimized for a narrow user group—reducing usability, acceptability, and trust among underrepresented populations (Green et al., 2022; Armstrong, 2024).

Previous efforts to address these disparities have primarily focused on enhancing recruitment strategies (Habibi et al., 2015) and increasing awareness among underrepresented groups. Initiatives have included targeted outreach programs, culturally tailored communication materials, and improved cultural competence training for researchers (Matshabane, 2021; Peebles et al., 2023). A smaller but growing body of work has begun to explore the value of community-led and participatory approaches (Güemes et al., 2025). These approaches emphasize collaboration with local communities not only as research participants, but as active partners in shaping study design, implementation, and dissemination. However, such approaches remain uncommon in neurotechnology, where most studies are still conducted in institutional settings with minimal cultural adaptation. As a result, there is a persistent gap in creating truly inclusive, community-centered research practices—underscoring the need for broader adoption of sustained, participatory models.

To address these issues, this study adopted a community-engaged, user-centered approach in partnership with Israac, a Somali community association based in Sheffield, UK. The research involved a series of community-based participatory workshops, focus group discussions, one-to-one interviews, and practical neuroimaging sessions using EEG, fNIRS, alongside engagement activities around non-invasive brain stimulation and prototype interaction. The study aimed to:

- Explore the social, cultural, and practical barriers influencing participation in brain research among Somali community members.
- Evaluate the acceptability and usability of EEG and fNIRS technologies from the participants' perspective.
- Assess the feasibility of collecting usable EEG and fNIRS data in non-laboratory community settings.

The central contribution of this study is to examine whether an inclusive, community-based approach can support participation in neurotechnology research while enabling the collection of usable neuroimaging data in a community setting.

To our knowledge, this is the first UK study to combine community-led workshops, on-site EEG/fNIRS data collection, and brain-stimulation engagement within a Somali community. By centring the lived experiences and insights of participants, this study provides a deeper understanding of the structural and cultural factors shaping engagement with neurotechnology and demonstrates the value of participatory approaches in developing more inclusive and context-sensitive research practices.

## 2 Methodology

### 2.1 Study design and research approach

**Figure 2:**
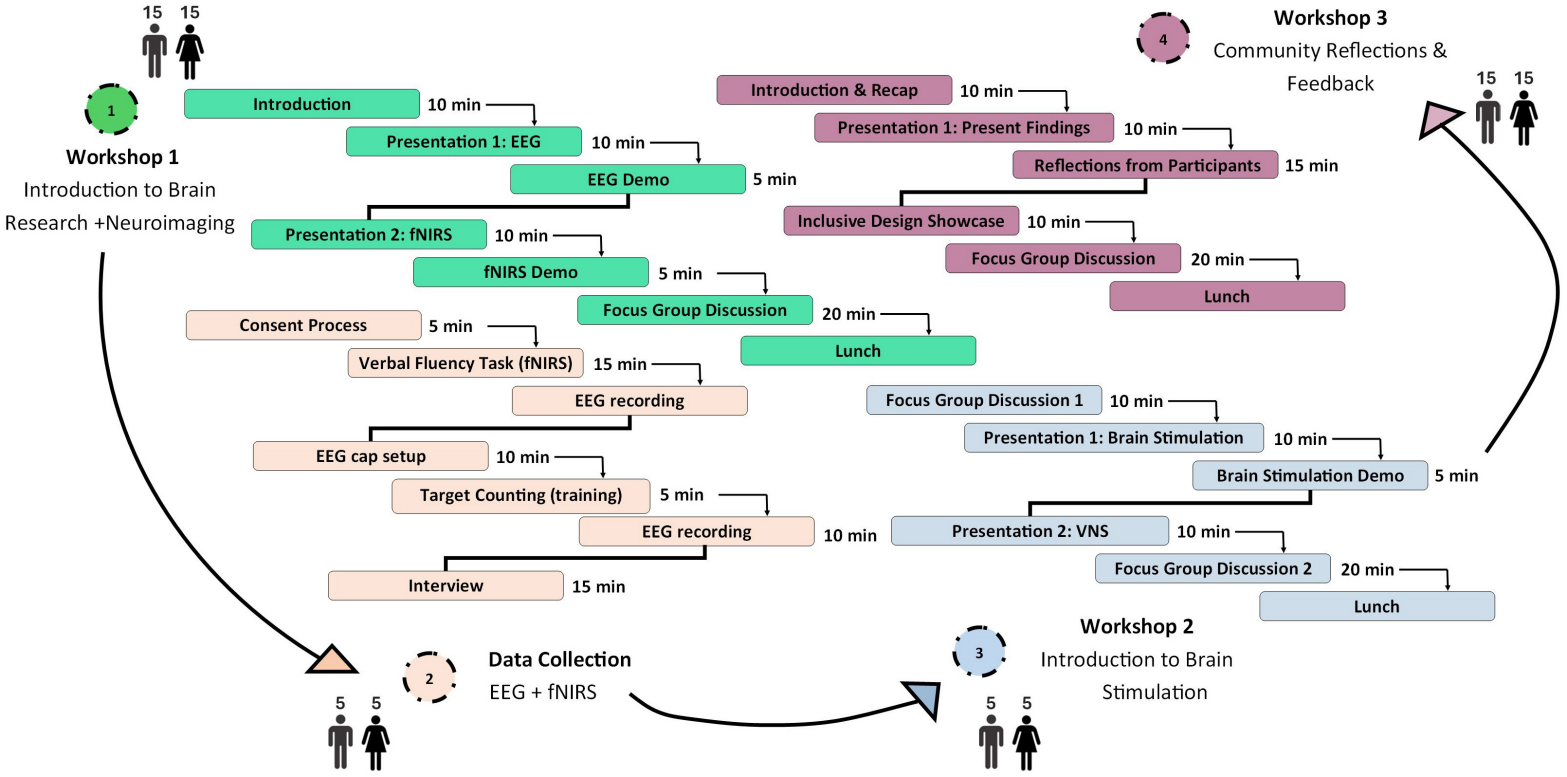
Flowchart illustrating the structure and sequence of the study's workshops and data collection session, all conducted in partnership with the ISRAAC Community Center. Workshop 1 introduced participants to brain research and non-invasive neuroimaging techniques (EEG and fNIRS), including live demonstrations and a focus group discussion. This was followed by a data collection session, where a subset of the participants completed cognitive tasks while EEG and fNIRS data were recorded, then took part in individual interviews and a short questionnaire. Workshop 2 focused on brain stimulation techniques—including tDCS, tACS, DBS, VNS, and FES—delivered through structured presentations, a demonstration, and two focus group discussions (held before and after the session). Workshop 3 was a reflection event, where participants reviewed preliminary findings, gave feedback, and were introduced to an inclusive EEG electrode design developed to accommodate diverse hair types. The flowchart highlights the timing and progression of each activity throughout the study.

This study employed a multi-phase, community-based design grounded in user-centered principles. The overall structure consisted of three workshops, sequenced alongside individual neuroimaging sessions, as outlined in Figure 2. This design aimed to build trust, establish cultural relevance, and iteratively increase participant engagement.

The study began with Workshop 1 held in November 2024. This session introduced core neuroimaging concepts and procedures through live demonstrations of EEG and fNIRS. It served as an entry point for participants to explore the technologies, share their initial perceptions, raise concerns, and engage in dialogue around trust and lived experiences related to brain technologies and brain research.

Following this, a subset of participants volunteered for individual neuroimaging sessions—conducted between November and December 2024—in which both EEG and fNIRS data were recorded in a community setting. These sessions aimed to evaluate the feasibility of collecting neuroimaging data outside clinical or laboratory environments. Immediately after each session, participants took part in brief follow-up interviews to share their perceptions of the procedures and overall acceptability of the research.

Building on the insights gathered, Workshop 2 was held in February 2025. Participants were introduced to brain stimulation techniques, such as Deep Brain Stimulation (DBS), transcranial Direct Current Stimulation (tDCS), as well as nerve or muscle stimulation methods such as Functional Electrical Stimulation (FES) and vagus nerve stimulation (VNS), through presentations and a live demonstration. Discussions focused on perceptions of safety, ethical concerns, and the cultural relevance of these emerging technologies within the community context.

Finally, Workshop 3, also held in February 2025, functioned as a reflective feedback session. It allowed participants to hear about our preliminary findings, share their views on research inclusivity and practices, and evaluate an EEG headset prototype that was developed in response to their earlier feedback.

Throughout the study, the researchers collected written notes of participants' responses separately, alongside their own observations of participant impressions and any challenges experienced during the workshops and neuroimaging sessions.

After each workshop, the research team and community members shared a communal lunch featuring culturally familiar dishes cooked by members of the community. These informal meals fostered a relaxed, welcoming atmosphere and played a valuable role in building trust and strengthening relationships over the course of the project.

### 2.2 Participant recruitment and demographics

This study received ethical approval from the University of Sheffield, School of Electrical and Electronic Engineering Research Ethics Committee. All procedures were conducted in accordance with institutional guidelines for research involving human participants.

Participants were adults (aged 18 years and older) of Somali origin residing in Sheffield. This group was targeted to center the voices of a population historically underrepresented in neurotechnology research and to uncover perspectives often absent from the development, application, and assessment of such technologies (Armstrong, 2024; Brown et al., 2023; Hall et al., 2022).

Recruitment was conducted in collaboration with the Israac Somali Community Association, a well-established local organization embedded within the community^1^. Two trained community champions—one man and one woman—played a key role in the recruitment process. As trusted members of the community, they helped identify and invite potential participants, sharing study information through familiar social networks and community gatherings. Their involvement supported culturally sensitive engagement and helped foster trust between the research team and the wider community. This recruitment approach was intentionally adopted to facilitate engagement with individuals who may be less likely to participate through conventional recruitment methods, in response to known barriers to participation in neurotechnology research among underrepresented groups.

Thirty individuals, equally divided by gender, participated in different stages of the study. Recruitment aimed to include participants across a range of ages while maintaining gender balance. Participants received financial compensation to acknowledge their time and contribution, including for each neuroimaging session attended, as well as for their participation in workshops and associated interviews.

Additional demographic information was collected only from participants who took part in the neuroimaging sessions; these details are reported in the corresponding results section. All participants received a plain-language explanation of the study's purpose, procedures, and potential risks or benefits. Written informed consent was obtained, with interpreters available for those who preferred to communicate in Somali. Participation was entirely voluntary, and all individuals were reminded of their right to withdraw at any point without consequence.

### 2.3 Community engagement and cultural sensitivity in study design

The study was designed with careful attention to cultural norms and sensitivities specific to the Somali community. Throughout both planning and implementation phases, the research team worked closely with community champions from the Israac Center to ensure all research activities were respectful, contextually appropriate, and aligned with participants' expectations.

Prior to each workshop and data collection session, study materials—including agendas, information sheets, and discussion prompts—were reviewed by community champions. Their feedback informed adaptations to language, content, and delivery style, ensuring that materials were culturally accessible and clear. Gender preferences and privacy needs were also taken into account; this included offering gender-specific group discussions where appropriate and assigning facilitators and interpreters matched by gender to support participant comfort.

Workshop and data collection schedules were arranged flexibly to accommodate participants' availability. Religious practices were respected throughout: when session times coincided with prayer or other religious obligations, appropriate breaks were provided to allow participants to perform their practices without disruption or discomfort. In addition, scheduling considerations included avoiding sessions during the fasting month and ensuring that workshop and recording times did not conflict with school runs, thereby supporting parents in fulfilling their caregiving responsibilities.

All personal data was anonymized, and any identifiable information was securely stored and handled in accordance with institutional ethical guidelines. Participant payment was reviewed with community input and aligned with National Institute for Health and Care Research (NIHR) guidelines to ensure it was appropriate and acceptable.

### 2.4 Research phases

#### 2.4.1 Workshop 1: Introduction to neurotechnology—Exploring perspectives, trust, and experience

The first workshop marked the initial point of contact between the research team and the wider community. It was designed to introduce participants to the core concepts of neuroimaging—specifically electroencephalography (EEG) and functional near-infrared spectroscopy (fNIRS)—and to gather community perspectives on brain research. The session included introductory presentations, live demonstrations, and structured group discussions.

The session began with the research team introducing themselves and providing a 10-min project overview, outlining the aims of the study, its funders, and the role of the brain in overall health. This included examples of neurological conditions such as Parkinson's disease and epilepsy. The overview was followed by two brief presentations: a 10-min introduction to EEG and a 10-minute introduction to fNIRS. Each presentation was accompanied by a live demonstration (approximately 5 mins), showing how the devices are prepared, fitted, and worn. These demonstrations gave participants the opportunity to observe the procedures first-hand and ask questions in real time.

Following the demonstrations, the participants joined structured group discussions facilitated by the trained researchers. Open-ended questions (see Table 1) invited participants to reflect on topics such as previous experiences with research, comfort with neuroimaging devices, perceived risks, and attitudes toward brain-related studies. Example questions included: “Have you participated in any medical or scientific studies before?,” “How do you feel about participating in research that involves brain activity monitoring, like EEG or fNIRS?,” and “How much trust do you have in medical or scientific research?”

**Table 1:** Discussion questions used across the three community workshops to guide conversations on experiences, perceptions, and concerns related to brain research and neurotechnology.

Workshop	Questions
Workshop 1	Have you ever taken part in a medical or scientific study before? If yes, what was the experience like?
	How do you feel about research that involves brain activity monitoring such as EEG or fNIRS?
	What would help you feel more comfortable or interested in joining a study like this?
	Do you think your community is generally interested in brain-related research? Why or why not?
	Have you or someone you know had a negative experience with research? How did that affect your view?
	How much trust do you have in scientific or medical research? What would help build more trust?
Workshop 2	Have you heard of brain stimulation technologies (e.g., tDCS, tACS, FES, and DBS) before?
	What is your first impression of using brain stimulation to treat conditions like epilepsy or Parkinson's?
	What kind of information or evidence would you need to feel comfortable with brain stimulation as a treatment?
	What are your thoughts about brain stimulation research overall?
	Would cultural, religious, or personal beliefs affect your willingness to try brain stimulation?
	What kind of support (e.g., from healthcare professionals, community leaders, or past patients) would help you feel more confident?
	What barriers might discourage you or others in your community from participating in brain stimulation studies?
	How important is it for you to trust the researchers and organizations running these studies? What affects that trust?
	How do you decide whether a medical or research institution is trustworthy?
	How can studies like this be made more accessible and welcoming for people from different backgrounds?
	What's the best way to inform your community about brain stimulation and related research?
	Whose voice do you trust most for information—doctors, researchers, community leaders, or people who have already taken part?
Workshop 3	What did you learn or discover during these workshops?
	What did you find most interesting or useful, and why?
	What could we improve in future neurotechnology workshops?
	Was anything confusing, uncomfortable, or less enjoyable? What would you change?
	Are there any other topics or hands-on activities you would like to see included?
	How did you feel during the sessions when you tried the EEG or fNIRS equipment?
	What could make the process more comfortable for others?
	What would help more people in your community feel encouraged to take part in studies like this?

Each set of questions was tailored to the focus of the respective workshop: prior research experiences and neuroimaging (Workshop 1), brain stimulation technologies (Workshop 2), and reflections on participation and future improvements (Workshop 3).

The workshop was open to a broad group of Somali community members as part of the study's inclusive recruitment strategy. It served both as a community engagement activity and as a preparatory step for those considering participation in the neuroimaging phase. The session also provided the research team with valuable insights into community expectations, concerns, and priorities, which helped shape subsequent parts of the study.

#### 2.4.2 Community-based brain data collection: hands-on learning and building confidence

The data collection phase was conducted with ten participants (five men and five women) who had previously attended Workshop 1. All sessions took place at the Israac Community Center. Each participant completed two neuroimaging recordings—using EEG and fNIRS—followed by an individual interview. During both recordings, participants engaged in cognitive tasks while their brain activity was recorded. This phase aimed to assess the technical feasibility of obtaining high-quality data in a community setting, while also exploring participants' perceptions of comfort, trust, and procedural clarity.

**a. fNIRS**

The fNIRS data was collected using a LUMO (Gowerlabs Ltd., UK) high-density continuous wave fNIRS system with 12 tiles placed in a frontal cortex array to record the hemodynamic changes during the tasks. Each tile contained three dual wavelength LED sources (1|2 = 735|850nm) and four photodiode detectors sampled at 12.5 Hz, forming 1,728 channels. Raw data was converted to concentration changes using the modified Beer-Lambert law (mBLL) in the MATLAB package Homer 2 (Huppert et al., 2009). The recording session was designed to capture participants' brain activity during semantic and phonemic verbal fluency tasks.

Following consent, participants provided demographic information on their sex, age, language background, hair color (Fisher-Saller scale), skin tone (Fitzpatrick, 1988), and ethnicity. One researcher then fitted the 12-tile frontal array fNIRS cap on the participant, measuring the head circumference and distance from the nasium for orientation. The signal saturation was visually assessed using the LUMO package interface. The cap was adjusted using a “fast-capping” technique (Yücel et al., 2024), until sufficient saturation was seen.

The fNIRS recording began following a verbal explanation of the verbal fluency tasks to the participant, with time to clarify the questions as needed. Participants were asked to refrain from speaking about non-task-related topics, moving, and adjusting the fNIRS cap when possible. These explanations were relayed and translated into Somali with the aid of a trained research champion as needed.

All participants completed six 60-s verbal fluency tasks in English or Somali, based on their preference, followed by a 30-s active rest period. The verbal fluency tasks included three category fluency tasks where they were asked to name as many items as possible from a category displayed on the screen: Animals, Supermarket items, and Jobs, followed by three letter fluency tasks where they named as many words as possible beginning with a specific letter: F, A, and S. Numbers and proper nouns were not permitted as responses during the task. All prompts (categories and letters) were shown visually on a laptop screen. During verbal fluency tasks, the category or letter target was verbally translated if requested at the beginning of the study. The words produced during each trial were recorded by the researcher or community champion for later analysis.

During resting trials, a fixation cross was presented for 30 s, and participants were asked to repeat the alphabet to measure activity during a speaking task without cognitive effort. During recordings from the first three participants, we found that asking participants to repeat the alphabet was not linguistically appropriate, as they were not sufficiently familiar with the English alphabet in a way that would mimic rote-learned speech, that is, speech that would not require thinking. As a result, this prompt was changed to reciting the days of the week starting with the fourth participant.

**b. EEG**

EEG data were recorded using the Enobio8 headset (Neuroelectrics) at a sampling rate of 500 Hz. Eight gel-based electrodes were positioned according to the international 10–20 system at Fz, Cz, Pz, Oz, C3, C4, P3, and P4 to ensure high-quality signal acquisition. The reference electrode was placed on the right earlobe. However, for four participants where a stable earlobe signal was difficult to obtain, a sticky reference electrode was instead placed on the mastoid area.

The recording session was designed to capture participants' brain responses during a cognitive task involving visual attention and mental counting. At the start of the session, participants were briefly introduced to EEG by observing their brain activity in real time. They were encouraged to blink, move their hands, and look around, allowing them to see how these movements created artifacts in the EEG signal. This familiarization helped improve their understanding of the system and encouraged cooperation, particularly in minimizing movement during the main task.

Before the experiment began, each participant completed a brief training session to ensure they understood the task and felt comfortable with the procedure. This step helped reduce confusion during the main recording. The experiment employed a visual three-stimulus oddball paradigm. In each block, participants silently counted how many times a specific target animal appeared in a sequence of different animal images. Each sequence included One target animal, One distractor animal with similar features (e.g., a hippo if the target was a rhino), and Four irrelevant animals (e.g., a panda). Participants were instructed to focus only on the target animal and ignore all others.

Each participant completed seven experimental blocks, each with a different target animal. At the start of each block, instructions appeared on the screen, which participants could read at their own pace. Once ready, they pressed the spacebar to begin. The name of the target animal was then displayed for 3 s, followed by a sequence of line-drawn animal images. Each image was shown for 200 ms, followed by a 600-ms black screen. All animal images appeared an equal number of times (12–14) in a randomized order. The presentation sequence was logged to align with stimulus markers during offline EEG analysis.

Between each of the seven blocks, participants were allowed to rest for as long as they needed to reduce fatigue and maintain focus throughout the session. To ensure accessibility and comprehension, all instructions and animal names were displayed in either English or Somali, depending on each participant's language preference. An interpreter was present to translate researcher instructions and comments in real time.

**c. Interviews**

Following the fNIRS and EEG recordings, participants were invited to reflect on their experiences through a brief questionnaire and a one-to-one interview (see Table 2). The questionnaire included Likert-scale items focusing on key areas such as perceived safety, physical comfort with the equipment, clarity of instructions, and trust in how their data would be managed. Some of these topics were also introduced at the beginning of the session to allow for comparison between participants' initial expectations and their post-session reflections.

**Table 2:** Interview and questionnaire items used during the post-data collection session.

Category	Questions
Safety perception	Did you have any concerns about the safety of EEG or fNIRS before participating? * Do you feel more reassured about their safety now? * If your perception changed, could you explain why?
Data handling and trust	Were you concerned about how your data would be collected and used? * Do you now feel confident that your data was handled properly? * What made you feel comfortable or uncomfortable about data handling?
Trust and initial thoughts	Did you have any other questions or concerns about EEG/fNIRS before the study? Have your views changed since taking part? What would help you feel more confident about research like this?
Motivation for participation	What motivated you to take part in this study? Please select all that apply: - To contribute to science and technology - To receive a voucher - Belief that it could help my community or society - Curiosity about how brain activity is measured - To try something new - Other (please specify):
Perceived benefit	Do you believe this research could be beneficial? Options: Yes—personally/for community/for society/No/Unsure If yes, in what ways?
Comfort with devices	How did you feel wearing the EEG cap and sensors? * How did you feel wearing the fNIRS sensors?*
Clarity of instructions	Were the instructions for EEG and fNIRS easy to understand?* Did any part of the setup or procedure feel unclear or uncomfortable?*
Cultural/religious considerations	Did the study respect your cultural, personal, or religious beliefs? Were there any specific beliefs that influenced your participation?
Participation factors	What helped you feel more comfortable participating? (Select all that apply): Options: Interpreter access, gender-matched researcher, private room, flexible schedule, feedback, support person, etc.
Future participation and advocacy	Would you take part in a study like this again?* Would you recommend this kind of study to your male or female friends?* If not, could you explain why?

These questions captured participants' perceptions of safety, data handling, device comfort, and cultural considerations after their direct experience with EEG and fNIRS. Both closed and open-ended responses were included to gather nuanced feedback. (*) refer to a 10-point Likert scale.

Individual interviews were then conducted to explore these themes in greater depth. The interviews took place in a private room and were facilitated by a researcher, supported by an interpreter of the same gender as the participant. The interviews were semi-structured and covered topics such as trust in researchers, understanding of the procedures, comfort during recordings, religious or cultural considerations, and perceived personal or community benefit of their participation in this research. Participants were also asked about their motivations for taking part, their willingness to participate again, and whether they would recommend similar studies to others in their community (see Table 2).

#### 2.4.3 Workshop 2: Introduction to brain stimulation—Perceptions, safety, and community views

The second workshop introduced participants to a range of brain and nerve stimulation technologies, including transcranial direct current stimulation (tDCS), transcranial alternating current stimulation (tACS), deep brain stimulation (DBS), vagus nerve stimulation (VNS), and functional electrical stimulation (FES). The session began with a 10-min focus group discussion designed to explore participants' existing knowledge, expectations, and any initial concerns about these techniques.

This was followed by a short presentation outlining the principles and clinical uses of both invasive and non-invasive stimulation methods. To support understanding, a 5-min live demonstration of tDCS and FES was conducted, where the devices were applied to a researcher in front of the group to help illustrate how the equipment works and what it might feel like in a real-world setting.

Next, a second presentation focused specifically on VNS, highlighting its therapeutic applications in neurological and psychiatric conditions. A follow-up focus group discussion, lasting around 20 mins, invited participants to reflect on the presentations and demonstrations, raise new questions, and share personal or cultural reactions to what they had seen.

As in the first workshop, the discussions centered on perceived safety, trust in researchers and devices, cultural and religious compatibility, and participants' willingness to engage in future studies involving brain stimulation.

#### 2.4.4 Workshop 3: Community reflections - shaping inclusive neurotechnology together

The third workshop served as a follow-up session for participants who had attended earlier workshops. Its purpose was to update them on the progress of the research and to share emerging outcomes shaped by their community's input. The session began with a 10-min introduction and recap, which revisited the study's aims and summarized key insights from previous sessions.

This was followed by a 10-min presentation of preliminary findings, including examples of EEG and fNIRS data collected in their community center. These were compared with typical recordings from controlled laboratory environments to highlight context-specific differences.

Next, a 15-min reflection segment invited participants to share their thoughts, feedback, questions, and personal experiences related to the study. This created a space for open dialogue and co-learning. The session then moved into a 10-minute Inclusive Design Showcase, where the research team presented a 3D-printed EEG electrode prototype. This prototype was developed in response to concerns raised in earlier workshops—particularly regarding comfort, hair texture, and signal quality. Participants were invited to handle the prototype and share their initial impressions.

The workshop concluded with a 20-min focus group discussion, which encouraged broader reflections on the research experience. Topics included suggestions for improving future workshops, ideas for additional activities, comfort during neuroimaging sessions, and ways to make participation more inclusive and accessible. These conversations were guided by open-ended questions listed in Table 1, and they offered valuable insights to inform future engagement and design strategies.

Finally, participants were invited to take part in an optional short video interview. This filming opportunity aimed to amplify their voices and help raise broader awareness about inclusive, community-led approaches to neuroscience and neurotechnology research.

### 2.5 Data analysis procedures

Responses from focus group discussions and individual interviews were analyzed using a qualitative thematic approach. Qualitative data were derived from detailed facilitator notes and structured session summaries recorded during and immediately after each workshop, rather than from audio recordings and verbatim transcripts. This approach was adopted to maintain a comfortable and culturally sensitive environment for participants. The research team reviewed all written notes and session summaries to identify recurring themes and participant concerns. A structured coding process based on the Gioia method (Magnani and Gioia, 2023) was used to organize participant quotes. Key themes included perceptions of safety, cultural and religious considerations, trust in researchers, and motivations for participating in neurotechnology research.

The analysis began with each researcher independently reviewing the session notes and identifying meaningful text segments. These segments were labeled using descriptive first-order codes that closely reflected participants' language. The codes were then grouped into higher-level second-order themes, which were further abstracted into aggregate dimensions following the Gioia methodology. Codes and themes were subsequently compared and refined through iterative discussion among the research team, and any disagreements were resolved through consensus. The team then convened to develop a shared set of themes, ensuring diverse participant perspectives were captured. As responses from male and female participants were collected separately—respecting the social norms of the Israac community—these were initially reviewed independently to identify gender-specific viewpoints, before being analyzed collectively to explore common themes across all participants.

Due to the small breakout group sizes during the workshops (five participants: one researcher), we opted to conduct the analysis manually to remain closely connected to participants' language and expressions. Questionnaire responses were reviewed alongside the researchers' discussion notes and interview summaries to provide additional context. All data were anonymized, and confidentiality was maintained throughout the analysis process.

In addition to the qualitative findings and Likert-scale questionnaire responses, EEG and fNIRS recordings collected during the community-based sessions were analyzed. Signal quality and neural response patterns were assessed and compared with data previously recorded in controlled lab environments. This comparison was used to evaluate the feasibility of conducting neuroimaging in community settings and to determine whether data of sufficient quality for meaningful analysis could be obtained outside traditional controlled research environments.

## 3 Findings

The findings of this study are organized into five subsections, each corresponding to a key research aim:

- **Understanding social and cultural barriers to participation:** four thematic categories emerged: (1) structural and socioeconomic barriers, (2) cultural and religious considerations, (3) awareness and perception of brain research, and (4) gender-specific differences in perceptions.
- **Perceived acceptability and usability of EEG and fNIRS technologies:** This subsection explores participants' experiences and perceptions of EEG and fNIRS procedures, including comfort, clarity of instructions, emotional responses, and trust in the technologies.
- **Evaluating the feasibility and data quality of community-based neuroimaging:** This subsection assesses the practical feasibility of collecting EEG and fNIRS data in a non-clinical community setting, with a focus on signal quality, environmental challenges, and overall suitability for inclusive research design.
- **Trust in brain research participation:** This theme explores how trust in researchers, institutions, and the research process is shaped, and identifies ways it can be strengthened.
- **Lessons and improvements for future practice:** This subsection outlines participant insights on improving engagement, communication, and delivery of community-based research.

### 3.1 Understanding social and cultural barriers to participation

#### 3.1.1 Awareness and perception of neuroimaging research

Participants across workshops and interviews demonstrated limited prior knowledge of neurotechnology and brain research. Most reported no previous exposure to devices such as EEG or fNIRS, and had not participated in related studies. For many, this was their first encounter with brain activity monitoring.

Initial reactions revealed widespread conceptual misunderstandings. For example, several participants associated neuroimaging with mind reading or brain interference. Questions such as “*Can it see my thoughts?*” and “*Will it affect my brain?*” were raised repeatedly. These assumptions were particularly common among individuals with no previous research experience and appeared to shape early perceptions of the technologies presented during the sessions. One participant reflected, “This was the first time I understood what this kind of research actually does.”

#### 3.1.2 Awareness and perception of brain stimulation research

In comparison to neuroimaging research, participants exhibited similarly limited awareness and mixed perceptions regarding brain stimulation technologies such as tDCS, FES, and DBS. Prior to the sessions, most had never encountered these concepts. Only a few individuals mentioned hearing about them through people they knew with conditions like epilepsy, while most had never come across the concept before. As one participant reflected, “I had seen someone using it before, but I did not know what it was for until now.” One participant shared that he had once seen someone using FES back in his home country but did not know what it was for until now. He reflected that it might have been a helpful option for a family member who passed away before receiving proper treatment. This example illustrated that while technologies like FES were available in their country of origin, a lack of awareness stood as a key barrier, preventing their use when it might have been beneficial.

The idea of applying electrical or magnetic stimulation to the brain raised fundamental questions about safety and permanence, especially where information was lacking or unclear. During the discussion, participants raised concerns about potential side effects and whether brain stimulation could cause harm or have long-term effects. Some participants expressed this uncertainty directly, questioning whether such interventions could “affect the brain permanently” or lead to unintended consequences. While 80% of those who had already taken part in the EEG or fNIRS sessions responded positively—citing their trust in the research team and the reassurance gained from experiencing non-invasive methods firsthand—around 20% remained hesitant. This group expressed concern that stimulation might alter brain function and stated they would only consider it in a clear treatment context, not for research purposes.

#### 3.1.3 Structural and socioeconomic barriers

Participants described a range of structural and socioeconomic factors that made it difficult for them to take part in research. One of the most common challenges was where the research took place. Many had only encountered studies in hospitals or academic institutions, which they described as formal, unfamiliar, and sometimes intimidating. In contrast, community venues—such as mosques or local centers—were seen as more approachable and comfortable. Several participants said they would have joined earlier if research had been held in spaces they already knew and trusted. As one participant explained, “Being here made it easier, because it is a place I already know and feel comfortable in.”

There was also a sense that researchers did not actively reach out to their communities. Participants described feeling overlooked, noting that studies were often “brought to others, not to us.” Written invitations, such as flyers or letters, were viewed as ineffective. Many felt that in-person contact would be a more respectful and effective way to build interest and trust.

Socioeconomic conditions also shaped how accessible research felt in practice. Some participants found that sessions were scheduled at times that clashed with caregiving responsibilities or school pick-up hours. Others mentioned the difficulty of traveling long distances, particularly when public transport was limited or unfamiliar. Even when individuals were interested, these everyday pressures made participation difficult to manage.

Language was another central issue. Several participants who did not speak English fluently explained that they often struggled to understand research materials or follow discussions. Interpreter support was viewed as essential—not only for basic comprehension, but also for enabling confident and independent engagement. Without it, some participants said they would have felt excluded or unsure about whether they could contribute meaningfully. Participants emphasized that clear communication and the ability to ask questions in their preferred language were critical to feeling included and able to contribute.

#### 3.1.4 Cultural and religious considerations in research participation

Cultural and religious values played a significant role in shaping participants' willingness to engage in neurotechnology research. Across workshops and interviews, participants—particularly women—emphasized the importance of gender matching and religious observance as essential preconditions for participation. For example, several women expressed discomfort at the idea of removing their hijab in front of male researchers, even in medical contexts. While some stated they would be willing to do so if absolutely necessary for treatment, many preferred to engage only with female researchers for tasks requiring hair exposure, such as EEG or fNIRS cap fitting.

Religious observance also shaped participation logistics. Attendees emphasized the need for flexible scheduling that accommodates prayer times, especially when sessions were held in community settings. Participants appreciated that the study provided breaks for prayer and created an environment that respected religious practices, which in turn fostered a greater sense of belonging and respect. These considerations were described not as preferences, but as necessary conditions for participation.

Importantly, the act of consulting with community champions and hosting sessions in familiar, trusted venues—such as the Israac Center—was seen as a demonstration of cultural sensitivity. These practices helped bridge the gap between academic research and community life, making participation feel more accessible and acceptable. Participants noted that such efforts made the research feel “more respectful” and aligned with their community values.

#### 3.1.5 Gender-specific differences in perceptions and engagement

While many concerns raised during the workshops were shared across participants, important gender-specific patterns emerged. These patterns are based on qualitative thematic interpretation rather than formal statistical comparison and should be interpreted accordingly, particularly in relation to comfort, cultural expectations, and willingness to engage with neurotechnology research.

Women were more likely to express concerns about modesty, privacy, and religious observance. The requirement to remove the hijab for EEG or fNIRS setup—especially in the presence of male researchers—was a major barrier. These concerns were not objections to the research itself but conditions that needed to be addressed for participation. Participants emphasized the importance of female researchers and private setup rooms to ensure cultural and religious respect. As one participant noted, “I would only feel comfortable if it was a female researcher.”

Men, by contrast, focused less on logistical or cultural barriers and more on the purpose and benefit of the research. They asked detailed questions about data access, brain recording methods, and potential community impact. Some also expressed mistrust toward institutions and funders, linking their hesitation to broader historical and political marginalization. Participants also raised questions about transparency, asking who was behind the research and how the data would be used.

Both groups called for greater transparency, clearer communication, and especially feedback on their own health data and study outcomes. However, the foundations of trust differed: for women, cultural safety and gender sensitivity were key; for men, accountability and clarity around how their data would be used and what benefits the research would deliver were more central.

### 3.2 Perceived acceptability and usability of EEG and fNIRS technologies

Participation in EEG/fNIRS was limited to a subset of participants and was voluntary, introducing potential self-selection bias. Those who proceeded were more likely to have developed sufficient trust and confidence through prior engagement, and, therefore, the neuroimaging findings reflect the perspectives of this subgroup rather than the broader workshop cohort. However, this staged participation approach was intentional, forming part of the study design to address low engagement in brain research by enabling a subset of community members to experience the technology directly and subsequently share their experiences with the wider group, thereby supporting trust-building and broader community understanding.

#### 3.2.1 Participant ratings on safety, comfort, and experience

**Figure 3:**
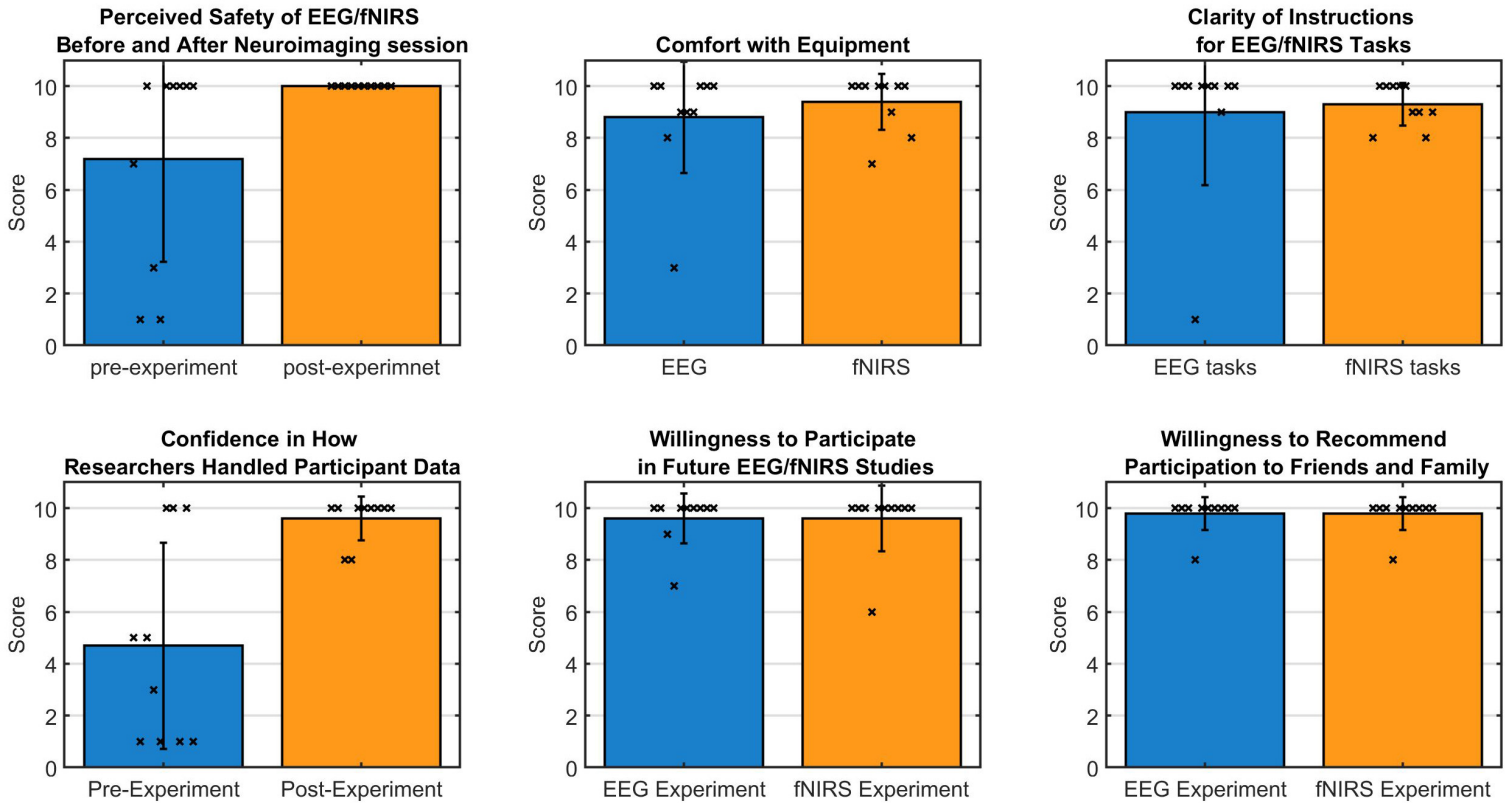
Ratings from 10 participants who took part in the EEG and fNIRS recording sessions, covering six key dimensions of neurotechnology usability and acceptability. Bars represent the mean score (±standard deviation) for EEG and fNIRS, with individual participant responses shown as black crosses. Categories include perceived safety, physical comfort, clarity of instructions, confidence in data handling, willingness to participate again, and likelihood of recommending the technology to others. Ratings were given on a 0–10 scale, with higher values reflecting more positive responses. Due to the small sample size, these ratings are presented as descriptive feedback to illustrate participant perceptions and are not intended to support inferential statistical conclusions.

Figure 3 presents ratings from the 10 participants who took part in the EEG and fNIRS recording sessions. They evaluated five practical aspects of each procedure—safety, physical comfort, clarity of task instructions, confidence in data handling, and willingness to repeat or recommend the experience—using a 10-point scale, where 10 indicates the most positive response. Given the small sample size, these ratings are presented as descriptive participant feedback to illustrate perceived experience and are not intended to support inferential statistical conclusions.

**a. Safety**. Perceived safety increased remarkably immediately after the recording sessions, compared to ratings given just before the session began. Initial concerns—particularly fears that the electrodes might “interfere with the brain”—were eased once the equipment was applied and its purpose clearly explained in plain language. These concerns were reflected in participants' accounts prior to engagement, with one participant noting that “Before the workshop, I was concerned and did not like trying new things.” By the end of the session, all participants gave the highest possible safety rating for both EEG and fNIRS procedures. This shift in perception was reinforced through direct experience, with participants reporting reassurance after the session, for example, “It was good—I did not feel anything.” Similarly, several participants expressed that their initial fears were largely based on misunderstanding, and that clear explanation combined with hands-on exposure played a key role in alleviating these concerns. Another participant noted, “When I saw how it was done and that nothing bad happened, I felt more relaxed about trying it myself.”

**b. Comfort and setup time**. Comfort scores were generally high, but they differed between EEG and fNIRS for a clear reason: the EEG cap took longer to fit. The cap needs gel and close contact at each electrode. This step is quick for people with short or loosely tied hair, but it proved slow for one participant with a tight natural high-density hairstyle. Parting the hair and pressing each electrode into place added several minutes, and the extra contact felt intrusive to that individual. This was also reflected in participants' feedback, with one participant noting that “The setup was time-consuming, which affected comfort.”

The longer setup time observed in some community sessions highlighted practical usability challenges during EEG preparation and electrode placement. In contrast, the fNIRS system used in this study was generally perceived as more comfortable, as it did not require gel application or repeated adjustments during setup.

**c. Task difficulty**. During the EEG session, participants performed a mental-counting task, quietly counting every time a target animal picture appeared on the screen. Most participants found this straightforward once the rule was explained. However, one participant rated the task clarity very low (1 out of 10). This was primarily due to technical difficulties during setup—his hair type was not compatible with the EEG cap, making it challenging to achieve a stable signal. The extended setup time and repeated physical adjustments made the participant uncomfortable, which negatively influenced his perception of the task, even though others found it easy. In comparison, The fNIRS session used a verbal-fluency task that some participants found harder, particularly when working in a second language. As a result, “instructions easy to follow” scores were slightly higher for EEG than for fNIRS, although both remained positive after we simplified the verbal task and added practice examples for the remaining seven participants.

**d. Data handling**. Concerns about data privacy followed a similar pattern to safety perceptions: confidence scores began in the mid-range but increased significantly after researchers explained that brain signals would be anonymized, securely stored, and never linked to participants' identities. This reassurance was provided twice—first in writing during the consent process (before the initial rating) and again verbally just before recording (after the first rating and before the second)—contributing to the marked rise in post-session confidence scores. This improvement in confidence was also reflected in participant feedback, with one participant stating that “Because it was computerized and password-protected, I felt more secure.” These responses suggest that clear, repeated communication played a critical role in building trust and reducing concerns around data privacy.

**e. Overall willingness**. Despite the isolated issues of hair management and task difficulty, willingness to repeat the session or recommend it to others stayed very high. Most participants, even those who had voiced early worries about comfort or complexity, said they would “happily do it again” and would tell friends the experience was safe and interesting.

#### 3.2.2 Challenges in data collection: community vs. laboratory environments

Both fNIRS and EEG procedures require researchers to place a cap on the participant's head, which can cause some discomfort due to the duration of the setup and physical contact involved. In the community setting, additional cultural and gender-related sensitivities emerged. Based on feedback from earlier workshops, the female participants were always paired with female researchers to respect cultural norms. However, it was initially assumed that male participants would be equally comfortable with male or female researchers. During data collection, some male participants expressed discomfort with physical contact from non-familial women, highlighting the need to consider gender-matching for all participants, not just women.

Another key distinction between laboratory and community-based data collection is environmental control. Community centers are designed to support social interaction, not scientific research. While this welcoming atmosphere is valuable for building trust and inclusivity, it also presented challenges—such as maintaining a quiet environment and preventing interruptions. During piloting, we encountered difficulties with background noise and unintended room entries. Rather than abandoning the community-based approach, we worked collaboratively with center staff and community members to adapt: identifying quieter times of day for data collection and posting clear signs in both English and Somali to minimize disruptions. This flexible approach allowed us to uphold data quality while respecting the community setting.

### 3.3 Evaluating the feasibility and data quality of community-based neuroimaging

We compared the quality of EEG and fNIRS data collected in the Israac Community Center with data acquired under similar task conditions in our laboratories at the University of Sheffield. Participant demographics for both environments are summarized in Table 3.

**Table 3:** Demographics of participants attending the Lab-based (fNIRS and EEG) and Community-based neuroimaging sessions.

Demographics		Lab-based fNIRS	Lab-based EEG	Community-based FNIRS/EEG
Number of participants		9	15	10
Age	Mean (SD)	35.8 (11.1)	25.4 (2.4)	46.7 (7.6)
Sex	Male	1	12	5
	Female	8	3	5
Hair color	No hair	–	–	4
	Gray	1	–	–
	Blonde	1	–	–
	Brown	4	7	–
	Black	3	8	6
Hair type	Straight (Type 1)	5	NC	1
	Wavy (Type 2)	2	NC	–
	Curly (Type 3)	2	NC	5
	Kinky (Type 4)	–	NC	–
	No hair	–	NC	4
Ethnicity	White	5	7	–
	Mixed Race Caribbean	1	–	–
	British Indian	1	–	–
	Asian	1	7	–
	Mixed South Asian-Arab	1	–	–
	Black African	–	–	10
	Arab	–	1	–
Skin pigmentation	Type I	2	NC	–
	Type II	2	NC	–
	Type III	4	NC	–
	Type IV	–	NC	–
	Type V	–	NC	–
	Type VI	1	NC	10

“NC” refers to “Not Collected.”

**Figure 4:**
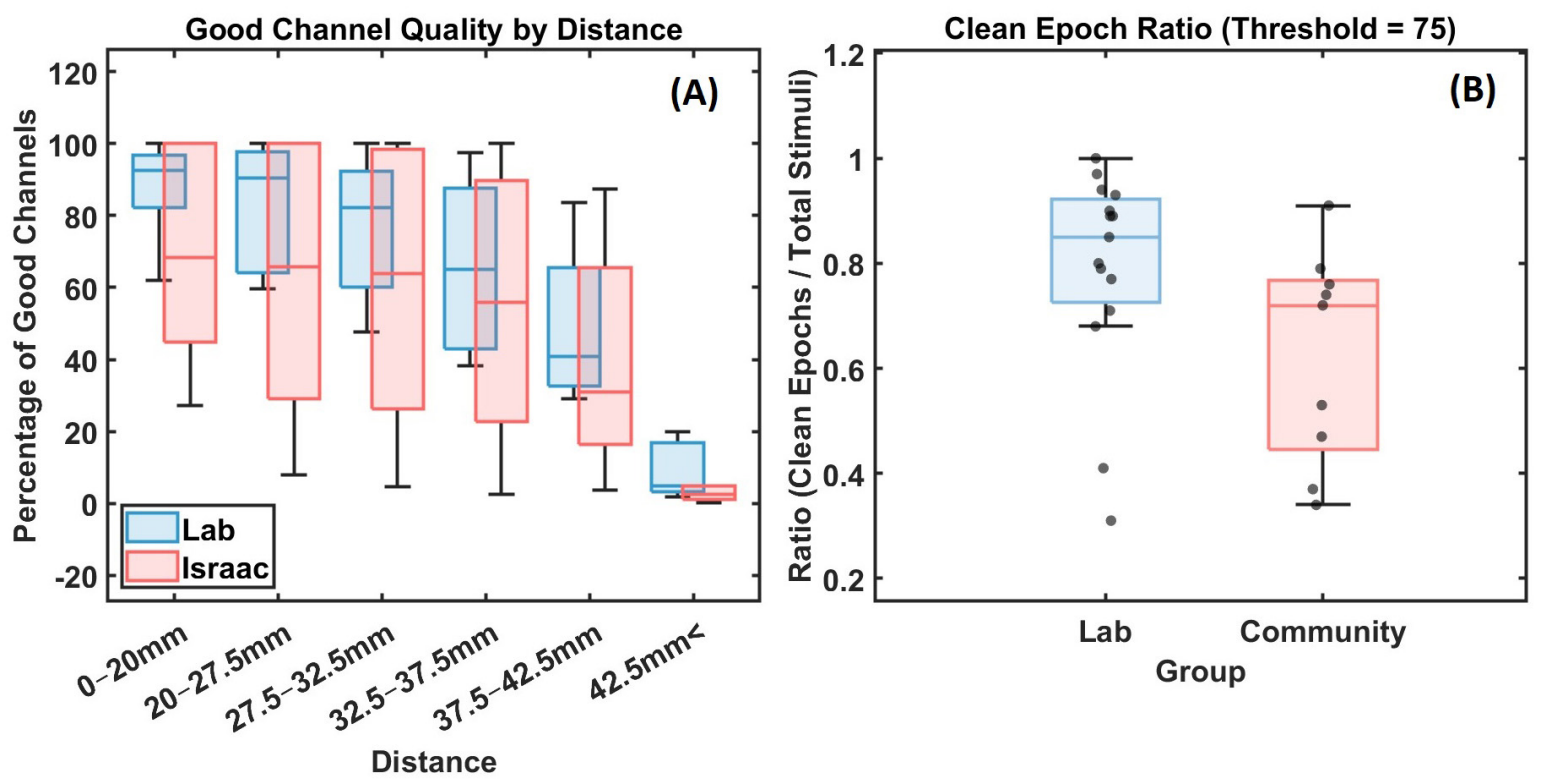
fNIRS and EEG Data quality comparison between recordings at the ISRAAC community center and in the lab. **(A)** Percentage of “Good” fNIRS source-detector channels at varying distances calculated using the DOTHUB_dataQualityCheck function in the DOTHUB MATLAB toolbox. **(B)** Clean epoch ratios from EEG recordings, based on a 75% amplitude-based threshold.

For fNIRS, we performed identical verbal fluency tasks across both sites. All participants were presented with the same stimuli across both sites. As lab data was collected after the Somali data collection, the adaptations made during piloting (i.e., changing alphabet repetition to weekday repetition during the rest task) was implemented in this data collection also. Data quality was assessed using the *DOTHUB_dataQualityCheck* function, which calculated the percentage of channels meeting pruning criteria (Range = [0, 2.5]; SNR > 12; SDrange = [0, 1e11]) at various source-detector distances (0–20 mm, 20–27.5 mm, 27.5–32.5 mm, 32.5–37.5 mm, 37.5–42.5 mm, >42.5 mm), as shown in Figure 4A.

To compare signal quality across groups, we created a generalized logistic mixed-effects regression model (GLMER), focusing on the count of good fNIRS channels, that is, those that passed key signal quality thresholds at each distance. Channels with a source-detector distance greater than 42.5 mm were excluded due to known reliability issues (Strangman et al., 2013). The model included fixed effects for cohort (community vs. laboratory), source-detector distance, and their interaction, as well as a random effect to account for repeated measurements within individuals. The GLMER model was specified as shown in Equation (1).


$$\begin{array}{l} \text{Count of Good Channels}\sim \text{Cohort}\times \text{Distance}\\ \;+\left(1|\text{ID}\right) \end{array}$$ (1)

This approach allowed us to test whether fNIRS signal quality differed between the two cohorts, whether it changed across different channel distances, and whether the effect of distance varied between settings, while adjusting for variability between participants.

A GLMER showed no significant difference in signal quality in the community group compared to the lab group, $\chi_{\left(1\right)}^{2}=0.17,$p = 0.68. Signal quality significantly declined with increasing source-detector distance, $\chi_{\left(4\right)}^{2}=644.00$, *p* < 0.001, with this decline more rapid in the Somali community, $\chi_{\left(4\right)}^{2}=13.18$, *p* = 0.01, where.

Given that many Somali participants had little or no hair, we repeated the analysis for only those participants with hair in the measured region. When controlling for hair presence, pattern of similar signal quality between the two groups remained, $\chi_{\left(1\right)}^{2}=0.33$, *p* = 0.57. The effect of distance remained significant, $\chi_{\left(4\right)}^{2}=499.27$, *p* < 0.001, and was no longer cohort-dependent, $\chi_{\left(4\right)}^{2}=8.69$, *p* = 0.07.

Following the fNIRS analysis, we compared EEG data quality between the community group (*n* = 9; one participant was excluded due to a technical issue during data acquisition, where event markers were not successfully recorded, preventing segmentation of the EEG data for analysis) and the laboratory group (*n* = 15) on comparable tasks. Other than minor differences in animal stimuli and language versions, all other conditions were identical. The same preprocessing pipeline was applied to both EEG datasets using MATLAB R2023b. EEG signals were bandpass filtered using a zero-phase 0.1–30 Hz filter, segmented into epochs from −100 to 1, 000 ms relative to stimulus onset, baseline-corrected using the −100 to 0 ms window, and subjected to artifact rejection using a 75 μV threshold. Data from all eight electrodes were included in the analysis. We then calculated the ratio of clean epochs to total epochs for each participant.

A boxplot was generated to visualize the distribution of clean epoch ratios, revealing a generally higher proportion of clean EEG data in the laboratory group compared to the community group (Figure 4B). For statistical comparison, we first assessed the normality of the clean epoch ratio distributions in both groups using the Shapiro–Wilk test. As neither distribution met the assumptions of normality, we conducted a Mann-Whitney U test (*p* = 0.053, *z*-statistic = 1.939, and effect size = 0.396) to determine whether the groups differed significantly. Our results suggest that EEG data quality, defined as the percentage of epochs preserved after artifact rejection, tended to be higher in the laboratory group than in the community group, although the difference did not reach statistical significance (*p* = 0.053). The effect size (*r* = 0.39) indicated a small-to-moderate group difference. Importantly, community-based EEG collection proved feasible, with an average of 70% of epochs retained compared to 84% in the laboratory, yielding data of sufficient quality for analysis. Although the study generated important insights into barriers, acceptability, and inclusive engagement strategies, the relatively small sample size limits the generalizability of the findings. In particular, the neuroimaging component should be interpreted as exploratory and feasibility-oriented rather than a statistically powered comparative analysis. Further studies involving larger and more diverse cohorts are needed to validate and extend these findings.

#### 3.3.1 Trust in brain research

Trust emerged as a central concern throughout the workshops and interviews, shaping how participants responded to the idea of engaging with neurotechnology research. The conversations revealed a range of factors that influenced how trust, or mistrust, was formed, often rooted in past experiences, collective memory, and long-standing concerns about exclusion.

A recurring point of tension was the lack of transparency around how research is organized and funded. Several participants openly questioned where the money for studies like this comes from and whether outside interests influence the process. When participants were unclear about who was involved or why the research was being conducted, suspicion often followed. Some asked directly who was behind the project and what researchers were hoping to gain. These questions weren't simply about curiosity; they reflected a deeper concern about whether the research would actually benefit their community or simply use their involvement for someone else's purposes, not dissimilar to the known mistreatment of minoritized communities seen throughout the history of research.

Concerns about data privacy were also widely shared. Participants expressed discomfort with being asked for personal details such as names, postcodes, and email addresses. Even when the request appeared routine, it raised questions about where the information would go, who would see it, and whether it might be shared without their knowledge. Some participants worried that the data could be used in ways that might affect their access to government benefits—particularly those related to low income or disability. This caution was also shaped by previous experiences, particularly in healthcare settings. Several participants spoke about feeling dismissed by their health providers, being misdiagnosed, not listened to, or treated with indifference. These encounters left a mark, and many carried those doubts into the research space.

The idea of being treated as a guinea pig surfaced multiple times. It captured something more than just a fear of procedures, it reflected a sense that research often happens *to* people rather than *with* them, especially in their community. This was echoed by one participant who noted, “I did not want to be approached by researchers as a ‘guinea pig.”' Finally, some participants recalled expressing interest in previous studies but never receiving a follow-up. This lack of continued contact left them feeling disregarded and reduced their willingness to participate in the future. It reinforced the impression that researchers were more focused on collecting data than building long-term relationships with the communities involved.

Trust in this research was not presumed. It was intentionally cultivated through clear guidelines and deliberate actions aimed at building strong and mutual trust. The process began with holding workshops at the familiar Israac community center, using gender matched facilitators, and providing local interpreters. These choices immediately signaled cultural sensitivity and comfort. Bringing the research into participants' own space was recognized as the first meaningful step toward trust. Another participant noted that trust was more likely when research was introduced through someone from their own community, rather than through unfamiliar organizations or external advertisements. Live demonstrations of EEG, fNIRS, and brain stimulation devices helped reduce fear and uncertainty. Participants were able to see and understand exactly what was involved. Clear reassurances about data confidentiality and the voluntary nature of participation addressed concerns around privacy and control.

Transparency played a key role. We explained the project's public funding, openly named our funders and institutions, and shared exactly how resources such as vouchers and equipment were used. This openness helped counter doubts and established credibility. The introduction of a three-dimensional printed EEG electrodes adapted for African style hair further demonstrated that participant concerns were not only heard but actively addressed. A final community reflection session created space for open dialogue and reinforced that participants were not merely subjects but collaborators shaping the process.

Alongside these structured efforts, we also learned from the community's own definitions and expectations of trust. Face-to-face interaction emerged as essential, as written materials alone were not persuasive—one participant remarked, “We need to see and touch.” Trust increased when community members tested the equipment themselves and shared their experiences, with several noting that others were waiting for their feedback before deciding to participate. One participant explained that she waited for a friend to take part in the neuroimaging session first, and only decided to participate after hearing that the experience was safe. It was further strengthened when research outcomes were presented clearly at the final workshop, and when participants saw tangible evidence of their impact—such as the three-dimensional EEG electrode prototype that had been designed directly from their earlier feedback. Some participants described this process as “breaking a barrier” in how they viewed research participation.

Sustained engagement was especially important. Participants valued that the research team visited multiple times. This was in contrast to past experiences with health providers who came once and never followed up, leaving them feeling used for data. Cultural and religious respect, such as accommodating prayer times and providing gender matched facilitators, was not seen as a courtesy but as a fundamental condition for trust. Trust was also closely tied to familiarity, with one participant noting that “I would not be willing to take part if a stranger approached me.”

### 3.4 Lessons and areas for growth

The workshops and data collection sessions revealed several strengths in the research design that contributed to successful community engagement, as well as areas where improvements could enhance future implementation.

A key strength was the provision of participant compensation, which was well received and reinforced a sense of value and respect for participants' time. The shared lunch experience, with food prepared by the community canteen, fostered a warm and informal atmosphere, strengthening trust and dialogue. Importantly, participants themselves noted a shift in attitudes within their community. They described how the workshops not only built trust with the research team but also enabled them to “break the wall” of fear and skepticism toward research among their peers. Additionally, participants consistently appreciated the respectful approach taken during the EEG and fNIRS setup, including the continuous request for consent before each procedure.

Despite these strengths, several challenges emerged. The first workshop was overcrowded, with around thirty participants. This made it difficult for interpreters to provide adequate support and also limited opportunities for individual contributions, as noted by the community champions. In addition, the large volume of information covered in a single session was overwhelming for some participants. Smaller, more focused sessions would likely have improved both clarity and engagement.

Data collection presented logistical challenges, particularly when quick turnarounds were required. The distance between the testing room and washing facilities made it difficult to clean EEG equipment efficiently, causing some sessions to run over time. In future planning, allocating additional setup time could improve efficiency. Background noise in the community space also interfered with concentration and data quality, underscoring the need for quieter, dedicated recording areas. This improved once clear reminder signs (e.g., “Quiet Please—Brain Recording in Progress”) were displayed throughout the venue to reduce disruption.

Technical and cultural sensitivities were also observed. One participant experienced discomfort during the EEG setup due to hairstyle compatibility, and the amount of physical contact required to adjust the cap was described as unpleasant. This emphasizes the importance of readiness, inclusive design, and minimizing setup time. While male participants initially reported flexibility regarding facilitator's gender, at least one expressed discomfort when fitted by a female researcher. This underscores the need to consistently offer gender-matched facilitators across all groups.

## 4 Discussion

The findings from this study highlight how deeply embedded social, cultural, and structural barriers continue to shape participation in neurotechnology research among underrepresented groups. These patterns align with previous health research that has identified such barriers as significant contributors to the underrepresentation of ethnic minorities in research more broadly. Through direct engagement with the Somali community in Sheffield, this study was able to explore these dynamics in greater depth.

A central theme that emerged was the limited awareness within the community about neurotechnology and research opportunities in general. For many participants, the absence of clear, accessible information reinforced the belief that research was irrelevant to their lives or communities. This was reflected in participant accounts, with some noting that they had previously encountered such technologies without understanding their purpose. This perception of irrelevance is not unique; previous studies have shown that ethnic minority groups often disengage from research when culturally appropriate and clearly communicated information is lacking (Spencer et al., 2022).

Closely linked to this was the issue of trust—both in the research itself and in the individuals or institutions presenting it. During the workshops, participants expressed concerns such as a lack of clarity around the study's aims, limited transparency about funding sources, and uncertainty about who was ultimately responsible for the project. As one participant noted, “Research is often brought to others, not to us,” highlighting a perceived disconnect between research initiatives and their community. These perspectives reflect broader concerns rooted in historical experiences of unethical research practices, particularly among marginalized populations. Such histories continue to shape the perception that research may offer little or no direct benefit to participants or communities and, in some cases, may even pose risks of harm, exploitation, or stigma (Bonevski et al., 2014). Nevertheless, participants also identified factors that could help foster trust, including long-term community relationships, the involvement of professionals from recognized institutions, and evidence that their participation would yield tangible outcomes.

Beyond trust, participants described a range of practical and structural challenges that affected their ability to participate. Many cited inflexible job schedules, caregiving responsibilities and limited financial resources as barriers that made it difficult to attend research sessions. These observations reflect common themes in existing literature on ethnic minority participation in the UK (Hussain-Gambles et al., 2004; Masood et al., 2024). Importantly, the location and format of research played a major role: traditional research venues such as hospitals or universities were often described as intimidating, while community-based spaces were seen as more accessible and welcoming.

These logistical barriers were compounded by language and communication challenges. Several participants who were not fluent in English found it difficult to understand written materials or follow complex discussions. In this context, the availability of interpreter support was crucial—not only for comprehension, but also for enabling participants to feel confident and respected within the study process.

Cultural and religious considerations, particularly among women, further shaped participants' willingness to engage. Female participants emphasized the importance of gender-matched researchers, privacy, and maintaining modesty—for example, being able to keep their hair covered during EEG or fNIRS sessions. This aligns with participant statements such as “I would only feel comfortable if it was a female researcher.” These reflections echo earlier findings that religious and cultural values can strongly influence women's research participation (Al Subeh and Alzoubi, 2020). While some male participants also acknowledged the importance of cultural sensitivity, their concerns tended to focus more on the goals and outcomes of the research rather than procedural factors.

Taken together, these qualitative observations suggest potentially different gender-related considerations influencing trust and participation. Female participants more frequently discussed issues relating to cultural and physical safety, whereas male participants more commonly emphasized accountability and potential community benefit. Both groups, however, expressed a shared desire for clearer communication, transparency, and the opportunity to provide feedback.

Despite these challenges, the study also illuminated clear enablers for participation and engagement. Participants consistently described the Israac Center as a comfortable and familiar space, and expressed appreciation for the use of culturally appropriate methods—such as face-to-face invitations, shared meals, and multilingual materials. Some participants described these approaches as “breaking a barrier” in how they viewed research participation. These aspects helped foster a sense of inclusion and community ownership. The positive responses to these efforts support the broader case for decentralized, community-led models in neuroscience research, particularly when working with historically excluded populations (George et al., 2014).

The hands-on experience with EEG and fNIRS devices also played a transformative role. Initial fears around safety, data misuse, or unfamiliar procedures were largely dispelled after participants had the chance to try the equipment. Post-session ratings confirmed improvements in perceived safety, trust, and willingness to participate again. These findings suggest that experiential learning, coupled with transparent and respectful communication, is a powerful tool in improving acceptability.

Alongside its positive outcomes, the study also revealed technical and design-related barriers. EEG caps were notably more difficult to fit on participants with natural or tightly coiled hair, leading to longer setup times and occasional discomfort. While this did not prevent participation, it underscored the need for more inclusive hardware design in neurotechnology—an issue that has been repeatedly noted in recent literature (Brown et al., 2023; Yücel et al., 2024).

### 4.1 Limitations

The study has several limitations. First, the neuroimaging component involved a small sample size (*n* = 10), which limits statistical power and the generalizability of quantitative findings. Second, comparisons between community-based and laboratory datasets were conducted using cohorts that were not demographically matched, introducing potential confounding factors such as age, sex, and phenotypic differences. Third, participation in neuroimaging sessions was voluntary, introducing the possibility of self-selection bias, whereby individuals who felt more comfortable or engaged were more likely to take part.

In addition, EEG preprocessing relied on a fixed amplitude-based artifact rejection threshold (±75 μV), and more advanced approaches such as Independent Component Analysis were not applied, which may have disproportionately affected noisier community recordings. These limitations should be considered when interpreting the findings, particularly with respect to comparisons between settings. Future work should incorporate larger, demographically matched samples, include relevant covariates such as age, and adopt more advanced preprocessing approaches to enable more robust evaluation.

## 5 Conclusion

This study explored the barriers limiting participation in neurotechnology research among African ethnic minority communities, focusing on individuals of Somali heritage in the UK. Through community-based workshops, semi-structured interviews and hands-on neuroimaging sessions, we identified core challenges, including low awareness, mistrust, logistical constraints, and cultural or religious considerations—particularly around privacy and gender dynamics.

Despite these barriers, we found strong evidence that inclusive, community-led approaches can foster trust and engagement. Participants responded positively to culturally adapted methods, familiar venues, and opportunities for direct interactions with the neurotechnologies. Post-session feedback reflected increased confidence, comfort, and willingness to participate again.

From a technical perspective, the study demonstrated the feasibility of collecting analyzable EEG and fNIRS data within a community setting, despite the practical and environmental challenges associated with conducting neuroimaging outside laboratory environments. These findings highlight the importance of decentralizing research and designing protocols that reflect participants' lived experiences.

Taken together, the study contributes novel insights into the barriers that restrict participation in neurotechnology research and offers evidence that community-based, inclusive approaches can enhance both scientific quality and social equity. Future research must continue to center the needs, voices, and experiences of excluded populations—not only as participants, but as co-designers of equitable neuroscience.

## Data Availability

The raw data supporting the conclusions of this article will be made available by the authors, without undue reservation.

## Footnotes

1
https://israac.org.uk/
